# Cardiac implantable electronic device infection due to *Mycobacterium* species: a case report and review of the literature

**DOI:** 10.1186/s13104-016-2221-1

**Published:** 2016-08-24

**Authors:** Bandar Al-Ghamdi, Hassan El Widaa, Maie Al Shahid, Mohammed Aladmawi, Jawaher Alotaibi, Aly Al Sanei, Magid Halim

**Affiliations:** 1 Heart Centre, King Faisal Specialist Hospital and Research Centre (KFSH&RC), MBC-16, PO Box 3354, Riyadh, 11211 Saudi Arabia; 2 Infectious Diseases, Department of Medicine, KFSH&RC, Riyadh, Saudi Arabia; 3 Alfaisal University, Riyadh, Saudi Arabia

**Keywords:** *Mycobacterium*, Pacemaker, Defibrillator, Infection, Endocarditis

## Abstract

**Background:**

Infection of cardiac implantable electronic devices is a serious cardiovascular disease and it is associated with a high mortality. *Mycobacterium* species may rarely cause cardiac implantable electronic devices infection.

**Case presentation:**

We are reporting a case of miliary tuberculosis in an Arab patient with dilated cardiomyopathy and a cardiac resynchronization therapy-defibrillator device that was complicated with infection of his cardiac resynchronization therapy-defibrillator device. To our knowledge, this is the third case in the literature with such a presentation and all patients died during the course of treatment. This underscores the importance of early diagnosis and management. We also performed a literature review of reported cases of cardiac implantable electronic devices infection related to *Mycobacterium* species.

**Conclusions:**

Cardiac implantable electronic devices infection due to *Mycobacterium* species is an uncommon but a well-known entity. Early diagnosis and prompt management may result in a better outcome.

## Background

Infection of cardiac implantable electronic devices (CIEDs) is a serious cardiovascular disease and it is associated with a high mortality. CIEDs infection may occur as a pocket infection or as infection on the leads with infective endocarditis. Cardiac device-related infective endocarditis (CDR-IE) may occur as a primary infection of the CIED system or as a secondary infection as a result of hematogenous seeding during a bacteremia secondary to a distant infected focus [1].

*Mycobacterium tuberculosis* (TB) is an infection that primarily affects the lungs, but it can involve any other organs and structures such as the kidney, spine, brain, and rarely the heart. TB disease can be fatal if not treated properly (http://www.cdc.gov).

Tuberculosis endocarditis (TBE) has been reported for many years with involvement of native or prosthetic valves mostly in cases of miliary TB [2]. *Mycobacterium* species are another uncommon but well-described pathogen in CIED infections [3].

We are reporting a case of miliary TB in an Arab patient with dilated cardiomyopathy and a cardiac resynchronization therapy-defibrillator (CRT-D) device. His condition was complicated with TB meningitis and a CDR-IE with vegetations on the leads. He underwent surgical removal of the CRT-D device and leads and a gram stain of the vegetations was positive for acid-fast bacilli (AFB). We also performed a literature review of reported cases with *Mycobacterium* species related CIEDs infection.

## Case presentation

A 50-year-old male Arab patient with non-ischemic dilated cardiomyopathy, biventricular failure with left ventricular ejection fraction (LVEF) of 20 %, moderate to severe mitral regurgitation and tricuspid regurgitation, left bundle branch block, and non-sustained ventricular tachycardia. Cardiac catheterization did not show any significant coronary artery disease. He underwent a CRT-D device implantation, as he was symptomatic with shortness of breath, New York Heart Association Functional Classification-II. He had no history of diabetes mellitus, hypertension, or dyslipidemia. He was on anti-heart failure medications and was on the heart transplantation list.

He presented to the Emergency Department 8 months after CRT-D device implantation with 2 months’ history of high-grade fever, chills, rigors, and weight loss. There was no history of concomitant respiratory, gastroenterology, cardiovascular, or neurologic localizing symptoms. There was no history of contact with febrile or TB patients. He was seen at another medical center and started on intravenous (IV) vancomycin and gentamicin for possible infective endocarditis involving his CRT-D device leads but with no improvement.

On presentation, he was looking chronically ill but not in acute distress. He was febrile with a temperature of 38.8 °C, but he was hemodynamically stable with a blood pressure of 107/57 mm Hg, and heart rate of 98 beats per minute. Oxygen saturation was 92–94 % on room air. Cardiovascular exam showed a jugular venous pressure (JVP) at 4 cm above the sternal angle; normal first and second heart sounds with a soft systolic murmur at left lower sternal border which was reported in his previous physical exam. The device site in the left upper chest was normal. There was no lower limb edema and no stigmata of infective endocarditis. Chest exam showed bilateral basal crepitations. Neurological exam was unremarkable.

Laboratory tests; CBC showed mild anemia, and thrombocytopenia with normal white blood count. Renal profile was normal and hepatic profile showed mild elevation of aspartate aminotransferase (AST) and total bilirubin, and low albumin. Cardiac enzymes showed mild elevation of Troponin T. Lactic acid was elevated. Laboratory test values are summarized in Table 1. The 12 lead electrocardiogram showed sinus tachycardia at 106 beats per minute with atrial sensed and biventricular paced rhythm.

**Table 1:** Laboratory test results on admission

Test	Patient’s value	Normal range
CBC		
WBC	6.11 10^9^/L	3.90–11.00
Hb	107 g/L	135–180
Hct	0.311 L/L	0.370–0.520
PLT	89 10^9^/L	155–435
Renal profile		
Urea	5.2 mmol/L	2.5–7.5
Creatinine	75 umol/L	64–115
e-GFR	>60 mL/min/1.73 m^2^	>60
Potassium	3.6 mmol/L	3.5–5.0
Sodium	124 mmol/L	134–147
Chloride	94 mmol/L	98–111
Liver function test		
ALT	15 U/L	10–45
AST	64.2 U/L	10–45
Albumin	25 g/L	32–48
Total bilirubin	29 umol/L	0.0–21.0
Glucose		
Random glucose	5.60 mmol/L	2.7–18.0
Cardiac enzymes		
CK	83 U/L	24–195
Troponin T	0.022 ug/L	0.01–0.10
Others		
CO_2_	18 mmol/L	22–31
Lactic acid	3.93 mmol/L	0.9–1.8

*CBC* Complete blood count; *WBC* White blood cell count; *Hb* Hemoglobin; *Hct* Hematocrit; *PLT* Platelets; *e*-*GFR* estimated Glomerular filtration rate; *CK* Creatine kinase; *CO*_2_ Carbon dioxide

**Fig. 1:**
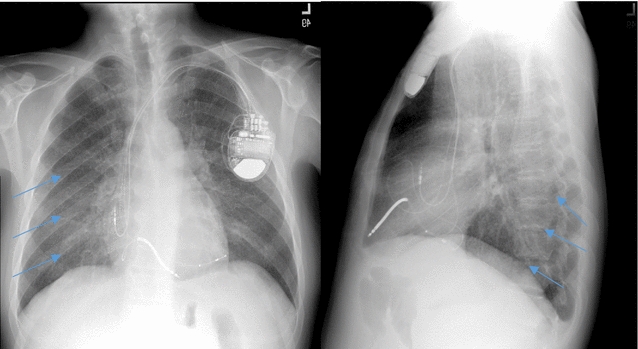
Posterior anterior and lateral chest X-ray showing diffuse miliary shadowing in both lungs (*Arrows*). The cardiac resynchronization therapy-defibrillator device and leads in place

The chest X-ray showed a diffuse miliary shadowing in both lungs suggestive of tuberculosis. There was mild ground-glass attenuation, especially in the right para-cardiac region. The heart was normal in size with CRT-D leads in place. There was no pleural effusion seen (Fig. 1).

**Fig. 2:**
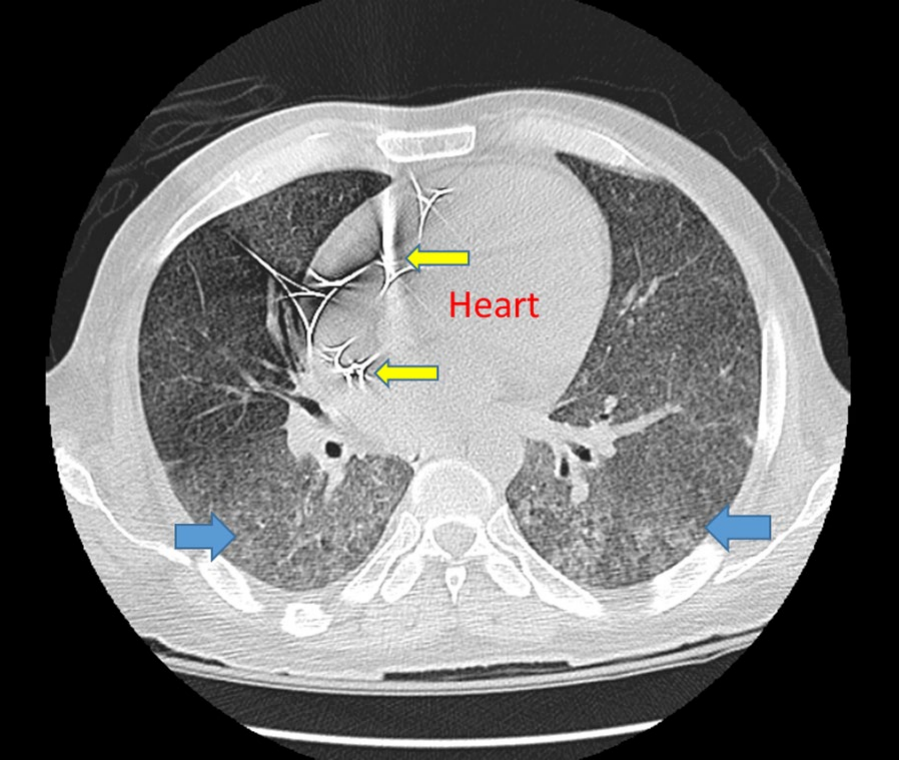
Computed tomography (CT) chest axial plane showing diffuse randomly distributed ground-glass nodules (*blue arrows*) involving bilateral lungs consistent with military tuberculosis. Implantable cardioverter defibrillator leads seen (*yellow arrows*)

The high-resolution computed tomography (CT) chest was consistent with miliary TB. There was no axillary, hilar or mediastinal lymphadenopathy (Fig. 2).

Based on the clinical presentation, the chest X-ray and CT chest findings, he was started on first-line anti-TB therapy in the form of rifampin 600 mg orally once daily, ethambutol 1200 mg orally once daily, pyrazinamide 2000 mg orally once daily, and isoniazid (isonicotinylhydrazide or INH) 300 mg orally once daily, with the addition of vitamin B6 25 mg orally once daily, and prednisone 60 mg orally once daily.

The trans-thoracic echocardiogram showed moderately dilated LV with severe global hypokinesis and severely reduced function (LVEF <25 %).The CRT-D device leads were seen in the right atrium and right ventricle. There were no obvious masses on the leads or on any of the cardiac valves to suggest vegetations. There was mild to moderate mitral regurgitation. The right ventricular systolic pressure was elevated at 30–40 mmHg. There was no pericardial effusion.

**Fig. 3:**
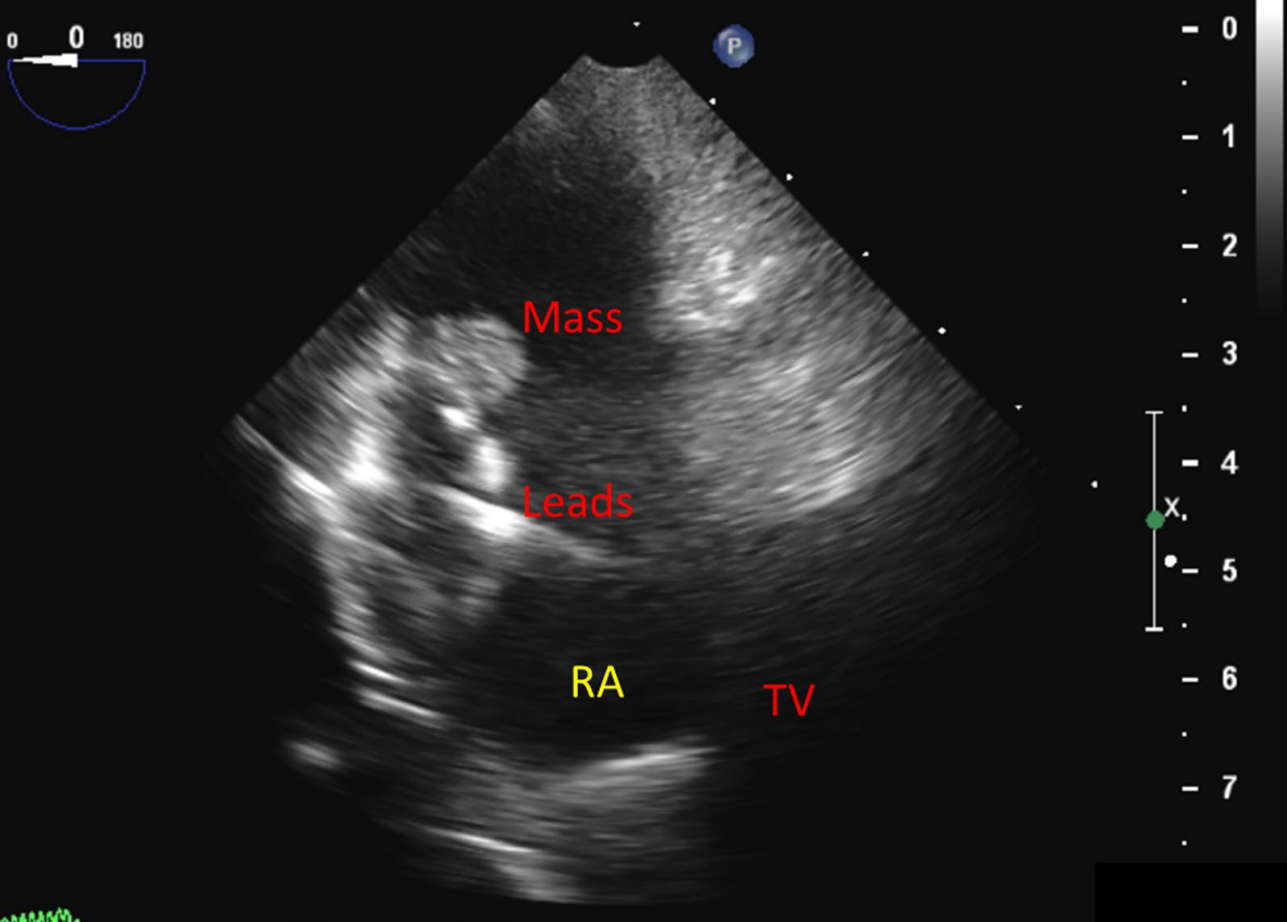
Transesophageal echocardiogram at mid esophageal level showing mass in the right atrium attached to the lead

The transesophageal echocardiogram was performed, and it showed a mass in the superior vena cava (SVC) around the CRT-D device leads, extending into the right atrium (RA). A thin flickering structure consistent with a remnant of a Eustachian valve or Chiari network was also noted. No masses were seen on them to suggest free moving vegetations on other parts of the leads, but some thickening was seen on one segment of wire. It was very thin and could represent a fibrin/fibrous tissue deposit. No masses were seen on any of the four valves. A small pericardial effusion was seen and “tissue” lines in the visceral pericardium were seen which could be fibrin, a hematoma, or tissue related to tuberculosis pathology (Fig. 3).

The blood cultures and urine culture were all negative. The work up was negative for HACEK group (*Haemophilus* species, *Aggregatibacter* species, *Cardiobacterium hominis*, *Eikenella corrodens*, and *Kingella kingae*), and other bacterial and viral infection including human immunodeficiency virus (HIV) and respiratory viruses.

Bronchoscopy and bronchoalveolar lavage were performed, and pathology examination showed a necrotizing granulomatous inflammation and special stains for AFB were positive. The culture showed *Mycobacterium tuberculosis* and sensitivity came back as sensitive to all first line anti-TB therapy.

His clinical course was complicated by acute renal failure with an increase in serum creatinine to 351 μmol/L and urea to 37 mmol/L. It was felt that the cause of his renal impairment was due to cardio-renal syndrome. It improved gradually and he did not require hemodialysis. He also had confusion and was noted to have a tonic–clonic seizure disorder. He was intubated, put on mechanical ventilation and started on anti-seizure treatment with IV levetiracetam (keppra) and phenytoin. Electroencephalogram showed a severe diffuse encephalopathy with periodic epileptiform discharges and continuous slow activity, generalized (delta coma). CT brain showed a preserved gray-white matter differentiation with no evidence of acute established territorial infarction, intra-axial or extra-axial hemorrhage, hydrocephalus, mass effect, or midline shifting.

Lumbar puncture was performed and cerebrospinal fluid (CSF) analysis showed a pale yellow, slightly turbid fluid with labs of: RBC 110 (normally 0), WBC 300 × 10^9^/L (0–5 × 10^6^/L) with neutrophils 72, band 1, lymphocytes 22, and monocytes 5. Glucose was 0.12 mmol/L (2.2–3.90 mmol/L), and protein 3580 mg/L (150–450 mg/L). *Mycobacterium tuberculosis* complex DNA was positive and Mycobacterium tuberculosis complex was isolated from the CSF. These findings were consistent with TB meningitis. Furthermore, he had melena with a drop in his hemoglobin to 69 g/L. He required transfusion of two units of packed red blood cells (PRBC). Upper gastroenterology endoscopy was performed, and it revealed a duodenal ulcer, which was cauterized. His thrombocytopenia continued and platelets dropped further to 14 × 10^9^/L. Hematology service was involved and it was felt that his thrombocytopenia was secondary to his TB infection. Coombs test was positive and peripheral blood smear showed no significant schistocytes making thrombotic thrombocytopenic purpura (TTP) unlikely.

**Fig. 4:**
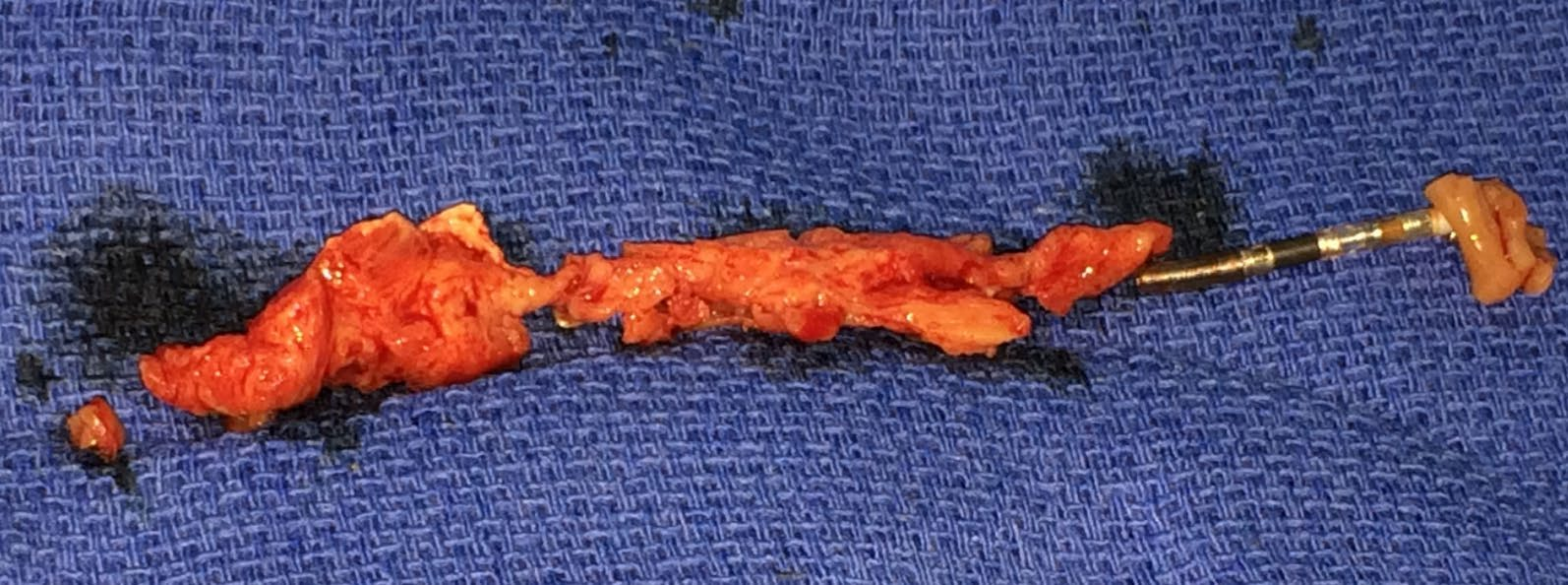
Vegetation on the lead

It was decided to remove his CRT-D device leads surgically due to the large size of the vegetations. Once he was relatively stable he was taken to the operating room and under general anesthesia a median sternotomy was performed and he was put on cardiopulmonary bypass machine. The RA was opened and the vegetation was noted to extend from the SVC to mid-RA (Fig. 4).

The vegetation was removed and sent for culture and sensitivity. The three leads were removed completely. Cardiopulmonary bypass was weaned off gradually with no difficulty. The patient tolerated the procedure and was transferred to the Cardiac Surgery Intensive Care Unit (CSICU) on inotropic support. The microbiology test of the vegetation revealed 10 colonies of *Mycobacterium tuberculosis* complex. In the CSICU, he was noted to have a high-grade fever and hemodynamic instability despite maximum inotropic support. He ultimately went into a multi-organ failure and died a few days later.

## Discussion

We are presenting a case of miliary tuberculosis (TB) with infective endocarditis involving cardiac resynchronization therapy device leads. We believe that this patient had a miliary TB that was complicated by infection of the CRT-D devices leads due hematogenous spread. There was no evidence of device pocket infection and miliary TB preceded the CDR-IE.

Infection of CIEDs is a serious cardiovascular disease which is associated with a high mortality. The incidence of CIEDs infection in a population-based study is 1.9 per 1000 device-years with a higher probability of infection after implantable cardioverter defibrillator (ICD) compared with permanent pacemaker (PPM) implantation [4]. The diagnosis and management of CIEDs infections are difficult [5].

Local device infection is defined as an infection limited to the pocket of the cardiac device and is clinically suspected in the presence of local signs of inflammation at the generator pocket, including warm site, erythema, fluctuance, wound dehiscence, erosion, tenderness or purulent drainage [6]. CDR-IE is defined as infection extending to the electrode leads, cardiac valve leaflets or endocardial surface [1]. As stated above our patient clearly had a CDR-IE secondary to hematogenous spread of the miliary TB.

Several factors have been associated with CIED infections, which may be divided firstly into patient factors such as renal failure, corticosteroid use, congestive heart failure, hematoma formation, diabetes mellitus, anticoagulation use, and fever within the 24 h before implantation [6–11]. Secondly, procedural factors such as the type of intervention [12, 13], device revisions, the site of intervention, the number of indwelling leads, the use of pre-procedural temporary pacing, failure to administer perioperative antimicrobial prophylaxis [14], and operator experience [11] all play a role.

Our patient had a CRT-D device with three leads which put him at higher risk for CIED infection.

In terms of microbiological causes of CDR-IE, Staphylococci especially Coagulase-Negative species (CoNS), accounts for 60–80 % of cases in most of the reported series [15, 16]. A variety of CoNS species have been described [6, 15]. *Corynebacterium* spp., *Propionibacterium acnes*, *Gram-negative bacilli* and *Candida* spp. are rarely identified as pathogens in CIED infection [6, 15].

CIED infection due to *Mycobacterium* species is an uncommon but a well-recognized entity.

Review of literature showed 25 case reports of CIED related infections due to *Mycobacterium* species [17–39], with two different *Mycobacteria* species in one patient [20], and three cases in one report [31]. Tables 2 and 3 show Mycobacterial infections of implanted pacemakers and ICDs respectively, as reported in literature. The infection was mostly pocket infection and rarely bacteremia with leads involvement. The infection occurred between 8 days and 20 years from the last device related procedure. It occurred with both transvenous [17, 18, 20–22, 24–27, 30, 31, 33–39] and epicardial device implantations [19, 23, 28, 29, 32]. *Mycobacterium tuberculosis* was reported in seven cases [18, 23, 28, 31, 36] with mostly pocket infection. In the two cases with miliary TB, both patients died. The first patient died in hospital after a prolonged admission due to renal and respiratory failure, and recurrent ventricular fibrillation [18]. The second patient died after discharge while awaiting re-implantation of ICD in spite of the fact he had been equipped with a LifeVest [36]. The late presentation, severe systemic involvement, and underlying severe heart failure were key risk factors in our patient’s ultimate outcome. *Mycobacterium* fortuitum was reported in nine cases [20–22, 24, 26, 27, 30, 38, 39] with pocket infection in five cases and pocket with lead or bacteremia infection in four cases. *Mycobacterium abscessus* was reported in two cases [19, 33] with pocket infection. *Mycobacterium* goodii was reported in two cases [25, 29] with pocket infection plus bacteremia in the first case. *Mycobacterium* avium complex [17], and *M. avium* intracellulare complex [32] in one case causing epicardial PM and ICD pocket infection respectively. Also it was reported with *M. chelonae* [20], *M. peregrinum* [34], and *M. phlei* [37]. These organisms are commonly found in the environment and likely contaminate the device or pocket at the time of insertion or during surgical manipulation. Reactivation of *M. tuberculosis* causing a CIED infection has also been identified in some of these case reports [23, 28, 31].

**Table 2:** Mycobacterial infections of implanted pacemakers as reported in literature

Author	Year	Age	Gender	Procedure/Type of device	Type of infection	Time from procedure	Organism	Clinical presentation	Management/Outcome
Amin et al. [17]	1991	21	F	PM generator change at the age of 20 years (PM at the age of 7 years)	Pocket and proximal leads	4 months	*M. avium* complex	Fever, pain, and swelling over the implant site	Device extracted by thoracotomy plus antimicrobial therapy (INH, RIF, ETH for 2 weeks)
Doherty et al. [18]	1996	70	F	VVI PM	Pocket	10 years	*M. tuberculosis* (Miliary TB)	Purulent discharge from the pocket and pyrexial illness	Died on her 31st hospital day due to renal and respiratory failure and recurrent VF
Cutay et al. [19]	1998	68	M	CABG surgery and epicardial pacing leads	Pocket and epicardial leads	20 years	*M. abscessus*	Erythema and discharge from the pocket site	Device extracted, and antimicrobial therapy (CLR, FOX, AMK for 5 weeks). Patient died about 1 month later due to ESRD
Verghese et al. [20]	1998	74	M	PM	Pocket	13 days	*M. chelonae*, and *M.fortuitum*	Fever, pain, purulent discharge from PM site	Device extracted, successful eradication with antimicrobial therapy (GEN, OFX for 1 month)
Sharma et al. [21]	2005	62	F	Biventricular PM	Pocket and lead endocarditis Bacteremia	9 months	*M. fortuitum*	Fever, chills, and pain at PM site	Device extracted, successful eradication with antimicrobial therapy (DOX, CIP for 6 months)
Hemmersbach-Miller et al. [22]	2005	72	M	PM in 2005	Pocket infection	2 weeks 1 year later	CoNS and *M. fortuitum* *M. fortuitum*	Abscess Subcutaneous nodules and chronic drainage	Device extracted, successful eradication with antimicrobial therapy (CIP then CIP, SXT and CLR then AMK and CIP) for 6 months
Hellwig et al. [23]	2005	8	–	Epicardial PM was implanted during CP-A anastomosis surgery New epicardial PM 5 weeks later	Pocket and epicardial leads	11 months 6 months	*M. tuberculosis* *M. tuberculosis*	subcutaneous abscess at PM site Fever, and inflammatory syndrome	Device extraction, successful eradication with antimicrobial therapy (RIF, INH, ETH, and PYR 2 months then isoniazid and rifampicin for another 7 months
Pastor et al. (Spanish) [24]	2006	80	M	DDD-R PM	Pocket infection Bacteremia	18 days (started 1 week before)	*M. fortuitum*	Fever, malaise, drowsiness, and purulent discharge	Device left in situ, successful eradication with antimicrobial therapy (CIP, CLR for 6 weeks)
Toda et al. (Japanese) [25]	2006	86	M	Generator change at 82 years	Pocket Bacteremia	4 years	*M. goodii*	Fever	Device extraction, successful eradication with antimicrobial therapy (INH, RIP and LVX)
Giannella et al. [26]	2007	84	F	Pacemaker upgrade	Pocket	1 month	*M. fortuitum*	Heart failure, fever, pain and erythema at PM site	Device extraction, successful eradication with antimicrobial therapy (LVX for 3 months)
Siu et al. [27]	2007	78	F	DDD PM then New DDD PM on right side	Pocket Endocarditis	3 months	*M. fortuitum*	Fever and erosion purulent discharge	Old and new devices extraction, successful eradication (LVX and CLR for 6 months)
Kestler et al. [28]	2009	80	F	CABG surgery and epicardial pacing wires	Pocket and leads	11 months	*M. tuberculosis*	Painful anterior epigastric mass	Drainage of abscess cavity and the wires were cut in the abdominal cavity (INH, RIF and ETH for 16 weeks, then INH and RFP total of 25 weeks)
Marchandin et al. [29]	2009	23	M	A mechanical double valve replacement with epicardial PM	Pocket infection	8-days	*M. goodii*	Fever then purulent discharge and wound dehiscence	Antimicrobial treatment (OFX and AMK then DOX) with recovery. PM was not removed
Al Soub et al. [30]	2009	15	F	PM generator change	Pocket, leads and myocardium Bacteremia	2 months	*M. fortuitum*	Discharge from surgical wound site and localized erythema and fever	Device extraction, successful eradication with antimicrobial therapy (DOX, and CIP for 6 months)
Kumar et al. [31]	2014	a.48 b.70 c.71	M M F	DDD PM VVI PM CRT-P(pulmonary tuberculosis 15 years back)	Pocket infection Pocket infection Pocket	15 months 18 months 60 months	*M. tuberculosis* *M. tuberculosis* *M. tuberculosis*	Subcutaneous abscess of the PM site Subcutaneous abscess at the PM site Large lump over the pacemaker site	Device left in situ antimicrobial therapy (INH,RIF,ETH for 3 months then INH and RIF for another 9 months) PM pocket debridement and antimicrobial Therapy (INH, RIF, ETH and PYR for 3 months then INH and RIF for another 9 months) PM device was explanted, antimicrobial Therapy (RIF, INH, ETH and PYR)

*CoNS*: a coagulase negative staphylococcus; *CP*-*A*: cavopulmonary arterial anastomosis; *CRT*-*P*: cardiac resynchronization therapy-pacemaker device; *ESRD*: end stage renal disease; *M*: Mycobacteria; *PM*: pacemaker; *TB*: Tuberculosis; *VF*: ventricular defibrillation

*Antimicrobial agents*: *AMK*: amikacin; *CIP*: ciprofloxacin; *CLR*: clarithromycin; *DOX*: doxycycline; *ETH*: ethambutol; *IPM*: imipenem; *LVX*: levofloxacin; *LZD*: linezolid; *MEM*: meropenem; *OFX*: ofloxacin; *PYR*: pyrazinamide; *RIF*: rifampin; *RFP*: rifapentine; *SXT*: trimethoprim-sulfamethoxazole

**Table 3:** Mycobacterial Infections of implanted cardioverter defibrillators as reported in literature

Author	Year	Age	Gender	Procedure/Type of device	Type of infection	Time from procedure	Organism	Clinical presentation	Management/Outcome
Katona et al. [32]	1992	31	F	ICD, with two epicardial patches and epicardial screw-in leads		1 year	*M. avium*-intracellulare	Pain and swelling at the abdominal insertion site of ICD	INH, RIF, and ETH and defibrillator leads were relocated to the other side of the abdomen 28 days later
Kessler et al. [33]	2004	53	F	ICD	Pocket	2 weeks	*M. abscessus*	Tenderness and brownish odorless discharge	Extraction of the device and CLR for 6 months
Short et al. [34]	2005	74	M	ICD	Pocket	6 weeks	*M. peregrinum*	Persistent erythema and a pustule	Extraction of the AICD and the leads. CIP and CLR for 6 weeks
Chrissoheris et al. [35]	2008	85	M	ICD (removal of PM and ICD implantation)	Pocket	Few days	*M. goodii*/*smegmatis*	Erythema, tenderness and fluctuance at the pocket site	extraction of the AICD and the leads and SXT for 8 weeks
Luckie et al. [36]	2010	67	M	Revision of CRT-D	Disseminated TB	Few months	*M. tuberculosis*	Fatigue, weight loss and anemia then pain around the ICD site, fluctuant swelling 6 months later	The ICD was explanted, and the patient discharged with a LifeVest and standard anti-tuberculous therapy Died at home
Karnam et al. [37]	2011	73	F	CRT-D	Pocket infection	1 months	*M. phlei*	serosanguinous discharge	The device and leads were explanted and prolonged antibiotic therapy SXT, DOX for 12 months)
Yuhning et al. [38]	2012	56	M	Upgrade to a CRT-D from PM then RA lead reposition	Pocket and leads	8 days	*M. fortuitum*	worsening pain and wound dehiscence and discharge	Antibacterial treatment (IPM, CLR and MOX; then MEM, LD and DOX) with device and lead extraction. Right MCA stroke, seizures, CoNS bacteremia, acute respiratory failure, and death
Shah et al. [39]	2012	78	F	ICD	Pocket and lead	–	*M. fortuitum*	Chest wall tenderness, fevers, chills, decreased appetite, weakness and weight loss	Antibacterial treatment with device and lead extraction. (SXT for 2 months)
Present case	2015	54	M	CRT-D	Leads	6 months	*M. tuberculosis*	Miliary TB	The device and leads were explanted and antibiotic therapy. Died due to multi-organ failure

*CoNS*: coagulase-negative staphylococci; *CRT*-*D*: cardiac resynchronization therapy-defibrillator device; *ICD*: implantable cardioverter defibrillator; *MCA*: middle cerebral artery

*Antimicrobial agents*: *AMK*: amikacin; *CIP*: ciprofloxacin; *CLR*: clarithromycin; *DOX*: doxycycline; *ETH*: ethambutol; *IPM*: imipenem; *LVX*: levofloxacin; *LZD*: linezolid; *MEM*: meropenem; *OFX*: ofloxacin; *PYR*: pyrazinamide; *RIF*: rifampin; *RFP*: rifapentine; *SXT*: trimethoprim-sulfamethoxazole

The widespread use of AFB staining and mycobacterial cultures has undoubtedly increased diagnostic accuracy [40]. Ziehl–Neelsen staining has a sensitivity of 50–80 % while that of blood culture is approximately 98 % [41, 42].

Early identification of the *Mycobacterium* species can provide predictable antimicrobial susceptibility patterns. The best chance of cure is obtained with a combination of at least two active antimicrobials given for a minimum of 4 weeks, plus removal of the CIEDs [43].

## Conclusion

CIEDs infection due to *Mycobacterium* species is an uncommon but a well-known entity. Early diagnosis and prompt management may result in a good outcome. In case of suspected military TB early initiation of anti-TB therapy is recommended while awaiting culture results.

## Abbreviations

°C: celsius
AFB: acid-fast bacilli
AST: aspartate aminotransferase
CDR-IE: cardiac device-related infective endocarditis
CIEDs: cardiac implantable electronic devices
CK: creatine kinase
CO_2_: carbon dioxide
CoNS: coagulase-negative species
CRT-D: cardiac resynchronization therapy-defibrillator
CSF: cerebrospinal fluid
CSICU: Cardiac Surgery Intensive Care Unit
CT: Computed Tomography
HACEK group: *Haemophilus* species, *Aggregatibacter* species, *Cardiobacterium hominis*, *Eikenella corrodens*, and *Kingella kingae*
HIV: human immunodeficiency virus
ICD: implantable cardioverter defibrillator
JVP: jugular venous pressure
LVEF: left ventricular ejection fraction
M: *Myobacterium*
PPM: permanent pacemaker
PRBC: packed red blood cells
RA: right atrium
SVC: superior vena cava
TB: tuberculosis
TBE: tuberculosis endocarditis
TTP: thrombotic thrombocytopenic purpura
